# Geometric Analysis and Experimental Studies of Hexachiral Structures

**DOI:** 10.3390/ma18184344

**Published:** 2025-09-17

**Authors:** Julian Plewa, Małgorzata Płońska, Kamil Feliksik, Grzegorz Junak

**Affiliations:** 1 Faculty of Science and Technology, Institute of Materials Engineering, University of Silesia in Katowice, 75 Pułku Piechoty Str., 41-500 Chorzów, Poland; malgorzata.plonska@us.edu.pl (M.P.); kamil.feliksik@us.edu.pl (K.F.); 2 Faculty of Materials Engineering, Silesian University of Technology, Krasińskiego 8, 40-019 Katowice, Poland; grzegorz.junak@polsl.pl

**Keywords:** chiral unit cells, Poisson’s ratio, auxetic structure

## Abstract

Chiral metamaterial structures exhibit auxetic properties—when subjected to stress, they either contract or expand in the given direction, while maintaining an asymmetric geometric effect—they cannot overlap with their mirror image. The unit cells of hexachiral structures take the form of cylindrical nodes with ligaments attached to them. Under the action of external compressive forces, the ligaments bend and coil around the nodes. This is accompanied by a negative Poisson’s ratio approaching minus one. In this case, it has been demonstrated both theoretically and experimentally that this value is independent of the degree of compression. In the course of geometric analysis, the value of Poisson’s ratio has been shown to depend on the number of unit cells in the structure, and with a large number of unit cells, it reaches the theoretical value of minus one. The experiments were conducted on structures assembled from printed nodes and ligaments. It has been demonstrated that, as a result of uniaxial compression, various parts of the structure undergo distinct deformations. However, structures subjected to multi-directional compression—as elastic energy reservoirs—also exhibited negative Poisson’s ratio values close to minus one, with their magnitude dependent on the degree of compression.

## 1. Introduction

**Figure 1:**
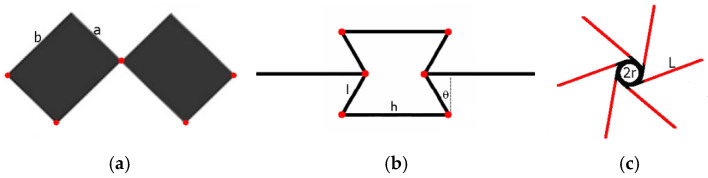
Unit cells marked in red indicate areas subject to bending, (**a**)—rotating rectangles structure, (**b**)—re-entrant structure, (**c**)—hexachiral structure.

Auxetic materials feature a cellular architecture and a negative Poisson’s ratio. This unique mechanical reaction is related to special geometries in the metamaterial’s cellular structure. Since the discovery of mechanical metamaterials, three designs of auxetics have been the basis for producing actual physical structures. They can be represented in the form of a network structure, where identical interconnected unit cells undergo deformation under the influence of external mechanical forces. The three structures in question have already acquired established names: structures of rotating polygons (squares, rectangles, etc.), re-entrant structures, and chiral structures. In the structure of rotating polygons, unit cells take the form of square or rectangular surfaces, or analogously shaped frames (Figure 1a). Re-entrant unit cell structures include short rods (struts) (Figure 1b), whereas chiral structures include cylindrical nodes—chiral cores with tangentially attached rods referred to as ligaments (nodes connected by tangential ligaments Figure 1c). The crucial condition for the occurrence of dimensional changes for all three mentioned types of structures is the bending of the connections: the connections of unit cells in structures of rotating polygons, the connections of struts in re-entrant structures, and the bending of entire ligaments in chiral structures. In the case of reversible deformation of structures, it involves dimensional changes within the elastic range of the material connections.

The above indicates that while in rotating polygons and re-entrant structures the joints bend, in chiral structures, this applies to the entire L ligaments.

The functioning of these metamaterial structures is thus based on the change in dimensions accompanying tension or compression, which allows for the determination of Poisson’s ratio. This physical quantity serves as a foundation for describing mechanical metamaterial structures with auxetic properties. Auxetic materials exhibit uniform dimensional changes and negative Poisson’s ratio values. The primary vulnerability of these three types of structures is their mechanical compliance, which leads to very small dimensional changes available within the elastic range, and their susceptibility to cracking and failure under either cyclic or large loads. Therefore, there is a problem of insufficient strength in the connections of the metamaterial structure’s elements, which is observable in the propagation of cracks under loading [1]. This is an evident obstacle for applications in the industrial environment [2]. Limiting the load magnitudes, most studies are conducted for a limited range of strains, i.e., in the small strain linear elastic region (usually 1% applied strain).

There is therefore a fundamental need for design approaches that enable the manufacture of structures that can function reliably when subjected to dimensional changes due to loading. Exceptions may include structures with purely protective functions that are subjected to destructive loads. Studies on impact behavior indicate a change in dimensions within the plastic range, as demonstrated, for example, in works related to energy absorption by chiral structures [3,4,5]. These structures, considered among the most popular auxetics and commonly known as “chiral honeycombs,” are thought to be particularly suited for providing protective functions [6].

In the case of chiral structures, numerous studies have been dedicated to their fabrication and improving their stability. However, it should be noted that most publications on chiral structures are based on simulation studies, which also yield ideas for potential applications.

Possible applications in lightweight constructions for the automotive industry, civil engineering, and aerospace have already been widely propagated [7,8,9,10,11]. The potential applications of chiral structures in medical equipment also constitute a significant area of interest [12,13], similar to their use in sensorics, where intelligent solutions are very feasible [14,15].

The majority of auxetic metastructures, including chiral ones, are currently being produced using additive manufacturing technologies [16,17,18] and laser melting [11,19]. This is also extensively discussed in review publications [12,20,21]. which also provide insight into other manufacturing techniques. The specified methods facilitate the creation of compliant structures, which are consequently homogeneous in terms of material composition. However, techniques based on assembling structures from individual chiral unit cells or their components do require a greater amount of effort, but they allow for the creation of more advanced structures. Cutting out chiral unit cell parts and using them to form structures has already been described in one study [3], while another one outlined the possibilities of designing modular and mass production of foldable auxetic metastructures [16]. Such chiral structures are implemented both in planar 2D form [16,17,18,19,20,21,22] as well as in 3D [22,23,24,25]; however, in an entirely chiral structure based on spheres, a significantly negative Poisson’s ratio has not yet been achieved [22].

In the present demonstration, mixed techniques for producing chiral structures were employed, assembling structures from printed polymer components. The assembly method facilitates the effective implementation of the given design.

The aim of this work is to study the compression process of selected chiral structures within the elastic range, focusing on identifying the occurrence of hysteresis in stress–strain curves in the tensile tests. This feature of metamaterial structures requires broader validation and further exploration. Here we present the experimental results and the observations derived from them.

In the present article, the axial mechanics of the general properties of chiral structures were investigated through analytical models and physical experiments. The results show that changes in the Poisson’s ratio are affected by the stiffness of the chiral system. The developed approach and the manufacturing process of chiral structures enable an engineering exploration of design solutions and the determination of conditions for their reliable operation.

### Geometrical Analysis of Hexachiral Structures

Chirality refers to an object characterized by non-overlapping mirror images. Therefore, the mirror images are two different molecules. Although chirality is a geometric feature, it is currently regarded as an important material property. Chiral metamaterials are also considered a special type of composite materials that exhibit, in particular cases, strong cross-interactions between the electric field and the magnetic field, resulting in extraordinary optical properties [26]. The interest in such composites arises from the demand, for instance, for the development of chiral drugs, sensors, catalysts, and photofunctional materials.

The field of metamaterials has become very broad and encompasses almost all aspects of solid materials, including optical, electrical, magnetic, thermal, and mechanical properties [27]. Chiral materials in applied physics include, among others, chiral nanostructures in optics [28], quasi-particles exhibiting the chiral magnetic effect [29], chiral phonons in ferroelectric materials [28], and graphene in edge state with the effect of giant magnetoresistance [28]. Chiral metamaterials are the subject of research involving the manipulation of optical activity to achieve magneto-optical effects [30,31] and applications in sensors [32,33].

Chirality in molecular and biological systems mainly involves DNA, which plays a crucial role in living organisms [34], as well as chiral particles that are useful for medicine and agriculture—enantiomers [35]. Thus, the chiral structures found in nature include, for instance, DNA, RNA, twisted flower petals and stems, climbing tendrils, and curling leaves, as well as chiral cellulose [36].

Chiral column chromatography in analytics [37] as well as chiral objects in catalytic reactions [38,39] are well-known areas of theoretical and experimental research in chemistry.

Original mechanical chiral metamaterial structures are a class of structures proposed by Wojciechowski [40] and later realized by Lakes [41]. According to their original work, based on the geometry of bent ligaments, it has been shown that a hexachiral structure theoretically exhibits a −1 Poisson’s ratio in the plane in two orthogonal directions. From a mathematical perspective, in two-dimensional space, a chiral structure has no axis of symmetry; whereas in three-dimensional space, it has no plane of symmetry or center of symmetry.

Although initially in chiral structures the ligaments must be tangent to the nodes, numerous modifications exhibit deviations both from this principle and partially from chirality itself [4]. Among auxetic metamaterial structures, one can distinguish trichiral, tetrachiral, and hexachiral structures, although anti-chiral structures have also been developed (with increased geometric versatility [42], as well as anti- and meta-chiral topologies for achieving desired properties [9,43]. These solutions exhibit significant differences compared to a typical chiral structure.

Structures known as 3D twisting mechanical metamaterials have achieved considerable popularity, produced by merging 2D structures with diagonal beams [44]. Research on torsional chiral structures is also quite extensive, due to their enhanced mechanical properties stemming from the coupling of compression-torsion and tension-torsion deformations [44].

Since the geometric design of chiral structures and theoretical investigation of their planar mechanical properties [41], there has been significant interest in the possibilities for their practical applications. A significant number of attempts have already been made to further develop or expand the scope of chiral auxetic structures and explore the potential engineering and social applications of these materials [7,8,9,10,11,12,13,14,15,44]. All of this research output is now available for application in the anticipated engineering projects. One can now highlight numerous breakthroughs achieved in simulations, yet also their lack in implementations. Thus, each new implementation may be the beginning of a broader application proposal.

**Figure 2:**
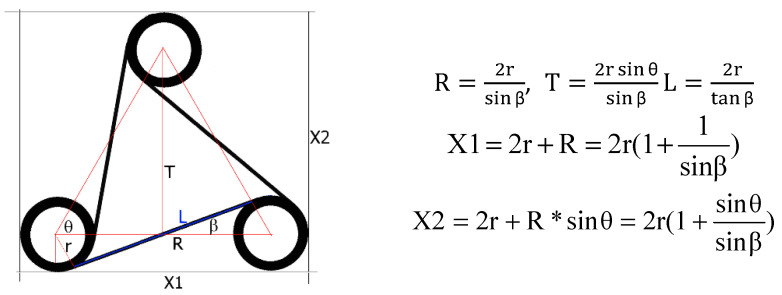
A part of a hexachiral structure with marked parameters, as well as the relationships for changes in the distance between the nodes (where r—radius of the circle at the node, R—distance between node centers, L—length of ligaments, T—distance between the center of the cylinder and the line connecting the opposite two cylinders; β—angle between the radial direction and the ligaments, angle between the line connecting the circle centers and the ligaments; θ—the angle between adjacent ligaments is π/3).

The hexagonal chiral structure depicted in Figure 2 consists of central cylinders (“nodes”) with six tangentially connected ligaments. In the presence of compressive forces, the ligaments bend, while the cylindrical nodes undergo rotational motion. In the context of flexibility, the strain energy is stored as “bending energy” in the ligament [11], which changes its shape to an S-form. The mechanism of deformation in chiral structures involves imparting a full wave shape to the ligaments. Theoretically, one can conceive a homogeneous continuum whose behavior in the elastic range allows for significant dimensional changes and a Poisson’s ratio of −1. Such ideal chiral networks are achieved by connecting the ribs (ligaments) of the round elements (nodes). The chiral hexagonal structure presented in Figure 2 consists of central elements in the form of cylindrical nodes with six tangentially connected ligaments. This hexachiral structure contains an ordered set of identical cylindrical elements (nodes) with a radius of r and connecting straight ligaments (or ribs) of length L. The condition for chirality is that the ligaments must be tangentially connected to the nodes. In this case, the angle between the adjacent ligaments is 60°.

The ideal hexachiral structure exhibits sixth-order rotational symmetry for which the theoretically determined Poisson’s ratio is minus one (−1) [41].

As far as the so-called engineering Poisson’s ratio is concerned [5], defined as the negative ratio of the relative linear strains, the initial and final states of the system are relevant. The theoretical value of −1 is obtained for changes in the distance ΔR between the centers of the cores and changes in height ΔT. However, considering the change in dimensions ΔX1 and ΔX2 of the outline of the structure, slightly different values of Poisson’s ratio are obtained.

The element of the hexachiral structure shown in Figure 2, when multiplied, serves as an example of an auxetic metamaterial continuum.

When choosing the radius of a cylindrical node r and the distance between the centers of the cylinders R, the initial angle of β is defined, which is the crucial part of the structure. Thus, the parameters r and R are referred to as “topology parameters” [45,46]. When compressed, the β angle value increases by Δβ, given the condition β + Δβ < 90°. The geometric parameter β defining the above relationship arises from the condition of tangency.

The parameters of the hexachiral structure are related to each other in the following way:

(1) $$\sin\beta =\frac{2r}{R},\;\tan\beta =\frac{2r}{L},\;\cos\beta =\frac{L}{R}$$

The chiral geometry is significantly altered by changes in r/R (or L/R = cos β), which in turn strongly affects the mechanical behavior [8].

The process of applying load to the structure is associated with a change in the β angle (Figure 2), whereas the change in its value is related to the value of the Poisson’s ratio. To trace the deformation of the structure and explain the mechanism of the dimensional changes, a theoretical illustration of their possible progression is provided below. This mechanism can be explored based on the analysis of two cylindrical nodes connected by a ligament.

**Figure 3:**
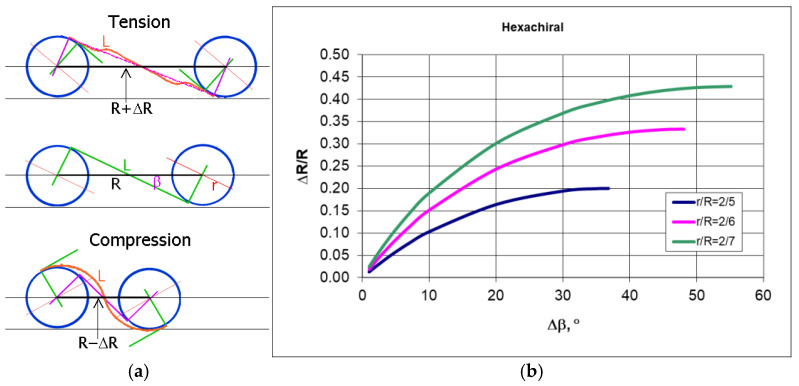
Changes in the shape of the ligaments in a chiral structure subjected to tension and compression—(**a**), change in the distance between the axes of the cylindrical nodes as a function of the increase in angle β in compression—(**b**).

**Figure 4:**
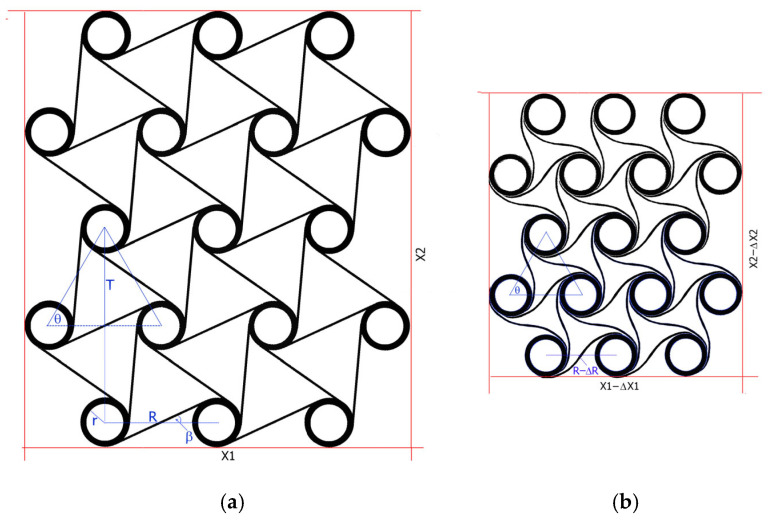
Example of a hexachiral structure (n = 4, m = 5) subjected to compression with uniform dimensional change, for r/R = 0.2223, initial state—(**a**), and after compression to ΔX1/X1 = 0.236—(**b**).

The configuration of two cylindrical nodes presented in Figure 3, connected with a ligament, is presented in three arrangements: stretched, initial, and compressed (top, middle, and bottom, respectively). The applied compressive load first causes bending of the ligaments into a “wavy” form, and subsequently induces a torque on the cylindrical nodes in such a way that they rotate in the plane. This rotation causes the wrapping of bent ligaments around each cylinder. At the same time, the cylinders move closer towards each other and can theoretically come into contact through the ligaments already wrapped around them. The wavy shape of the ligaments (also in Figure 4b) is a consequence of their bending and the effect of cylinder rotation caused by the load.

The auxetic effect in this type of chiral auxetic structure is achieved both by bending the ligaments and by their wrapping or unwrapping around the nodes under the applied load. It is evident that in compression, the angle β increases, as does the change in the distance between the cylinders ΔR, due to their movement towards each other—Figure 3b. The change in dimensions between the centers of the cylinders horizontally ΔR and vertically ΔT is accompanied by the compression of the structure.

Theoretically, in compression, this behavior is exhibited by all elements of the structure. Figure 4 shows a hexachiral structure (3-4-3-4-3) subjected to uniform deformation. Straight ligaments assume a wave-like shape, and the distances R between the centers of the cylinders change. For uniform deformation, the relative change in dimensions of the structure outline ΔX/X is close to the relative change in dimensions between the centers of the cylinders ΔR/R, as it is equal to ν = −0.9829.

In the hexachiral structure, each (internal) cylindrical node is connected to its nearest neighbors by eight ligaments. 17 unit cells combined into 5 chains (m = 5) lead to the twisting of chiral unit cells during compression and contraction of the whole 2D auxetic metamaterial structure—shown in Figure 4b. It is assumed that the value of the angle θ remains constant at 60°. Reversing the rotation of the chiral unit cells causes the chains to expand, which also leads to a negative Poisson’s ratio. The mechanism of dimensional changes in the demonstrated chiral structure involves bending and twisting. This coupling is caused by the chiral arrangement of the ligaments, which exert a reaction force system on the cylindrical nodes that are unbalanced in relation to this rotation. As a result of compression, the porosity of the structure significantly decreases, leading to an increase in its stiffness and density.

**Figure 5:**
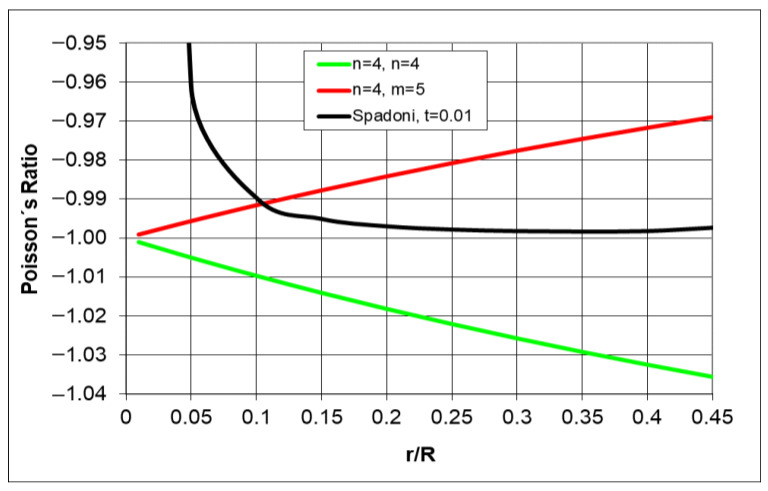
Comparison of the Poisson’s ratio values as a function of the parameter r/R for hexachiral structures.

For analogous hexachiral structures, the Poisson’s ratio depends on the geometry, and the smaller the ratio r/R (the longer the ligaments L), the closer the Poisson’s ratio gets to −1 (Figure 5). Here, the value of the Poisson’s ratio was calculated from the outline of the structure.

The relationship presented in Figure 5 for the hexachiral structure can be described by the following formula:

(2) $$\nu =-\frac{n-1}{(m-1)\sin\theta }\frac{\frac{2r}{R}+(m-1)\sin\theta }{\frac{2r}{R}+(n-1)}$$

where n i m—the count of the cells in the horizontal and vertical directions, r—radius of the cylinder, R—distance between the axes of adjacent cylinders, angle θ = 60° for a hexachiral structure.

One could easily demonstrate that for large numbers of unit cells, the value of Poisson’s ratio from Equation (2) reaches a minimum of minus one (−1), similarly to what is observed in practice for long ligaments. Poisson’s ratio is independent of the degree of strain and depends only on the geometric parameters r/R, given a specified number of unit cells.

From Equation (2), it follows that for an ideal hexachiral structure, Poisson’s ratio assumes negative values, which drop even further as the ligaments shorten (for n = m). It has also been shown that it is affected by the number of unit cells in the structure. This theoretical dependence indicates that the Poisson’s ratio value for a hexachiral structure does not depend significantly on the level of strain. In comparison, Figure 5 shows the course of the Poisson’s ratio changes according to the formula provided in another study [45]. It clearly shows a different trend, especially for small r/R values, which corresponds to long ligaments. However, Poisson’s ratio is a function of the parameter L (ligament length) and t (ligament thickness) as well as the β angle. The above study has also observed that the Poisson’s ratio depends on the geometry of the ligaments t/L and can equal −1 only for t/L = 0 [45].

The given formula (2) shows that Poisson’s ratio is determined by the value of the ratio r/R (assuming a constant ligament thickness). The ratio determines the geometry of the structure, due to the relationships as in the first Equation (1), showing that the structural parameters are interrelated: the radius of the cylindrical nodes r and the distance between the centers of the nodes R through the beta angle. Thus, the length of ligaments L is related to this ratio. It can also be added that the value of the ratio r/R varies from 0 to 0.5, while the corresponding ratio r/L varies from 0 to 1. In this case, the increase in the r/R ratio (shortening of the ligaments) pertains to structures with an equal number of unit cells in both horizontal and vertical directions (n = m).

**Figure 6:**
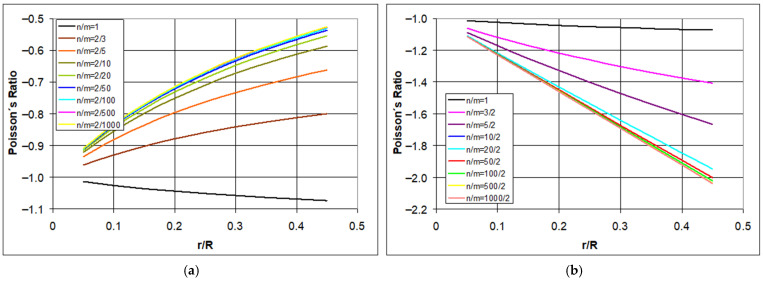
The relationship between the Poisson’s ratio and the r/R and n/m ratio where (**a**) for a variable number of rows m (2/m), and (**b**) for a variable number of columns n (n/2).

Taking into account the possibility of different numbers of unit cells in the structure, the changes in Poisson’s ratio were considered for different values of n/m. The new theoretical trajectories obtained are presented in Figure 6. The negative values of the Poisson’s ratio decrease for n/m < 1 (Figure 6a), whereas for n/m ≥ 1, they become more negative. This indicates that to achieve more negative values of the Poisson’s ratio, the hexachiral structure ought to be elongated horizontally.

From the presented graphs (Figure 6), it follows that the value of the Poisson’s ratio changes monotonically with the increase in the ratio r/R. These changes are relatively significant for small values of the ratio r/R, particularly for elongated ligaments. As the length of the ligaments decreases (increased ratio of r/R), changes in the Poisson’s ratio progress more smoothly.

However, as the number of chains increases (vertical growth), the value of the Poisson’s ratio becomes less negative, reaching approximately −0.5. Conversely, as the structure is stretched horizontally, the negative value of the Poisson’s ratio increases up to about −2.2. For structures with an equal number of unit cells in both horizontal and vertical directions (*n* = *m*), the value of the Poisson’s ratio is close to negative one and becomes increasingly negative as the ratio r/R increases, i.e., as the ligaments shorten.

Earlier studies have found that increasing the value of the ratio r/R influences a decrease in Poisson’s ratio, which thus becomes more negative [3].

The above findings, based on a geometric analysis of the hexachiral structure undergoing ideal dimensional changes, also indicate that the shortening of the ligaments (increase in the ratio r/R), meaning an increase in the construction angle β, may affect the reduction in the negative value of the Poisson’s ratio (Figure 6a), but only for the condition n/m < 1. The specific conclusion from these analyses is that the value of the Poisson’s ratio of ideal hexachiral structures does not depend on the magnitude of strain.

The change in the value of Poisson’s ratio as a function of the parameter r/R presented in Figure 6 corresponds to an idealized situation where the dimensional change during compression is uniform and does not depend on the degree of strain, the type of material, or the thickness of the ligaments. This idealized representation of the behavior of chiral structures has already been significantly adjusted, both by available simulation research and experimental work.

The presented considerations indicate that the value of the Poisson’s ratio determined based on the outline of the structure is different from −1, although it approaches that value for a large number of unit cells. However, calculating it based on changes in the distance between the axes of the cylindrical nodes and with a constant θ angle value, the theoretical value of the Poisson’s ratio equal to −1 is obtained. The two methods for determining Poisson’s ratio for hexachiral structures under loading demonstrate no influence of the degree of strain. In practice, however, considering all possible behavior of unit cells, one obtains very different values. The deviations from the theoretical value mainly arise from not maintaining a constant angle θ.

One study demonstrated that at high strains, the Poisson’s ratio varies considerably. This always leads to Poisson’s ratio exceeding −1 [45,46,47,48,49,50,51].

However, the change in the Poisson’s ratio due to changes in geometric parameters, including the dependence on the structural angle β (i.e., the ratio of r/R), has already been confirmed as a decreased auxetic effect at lower β angle values [47,48].

Another geometric parameter that affects the value of the Poisson’s ratio emphasized in the literature is the wall thickness t of the ligament [46,49,50].

However, for a physical hexachiral structure with specified geometric parameters (r/R and t/L), the value of the Poisson’s ratio is affected by local changes; during bending of the ligaments, when the β angle opens, or when the cylindrical nodes come closer to each other.

In general, typical chiral structures are prone to instability. This results from the strong anisotropy of such structures. Regular deformations under uniaxial stress occur only for small dimensional changes. The number of unit cells arranged in layers affects the deformation characteristics as well as the mechanical properties [52].

In the experiments, an attempt was made to demonstrate the real changes that hexachiral structures undergo when subjected to compression. The experiments were limited to a few selected structure types.

## 2. The Experiments

### 2.1. Sample Materials and Design

**Figure 7:**
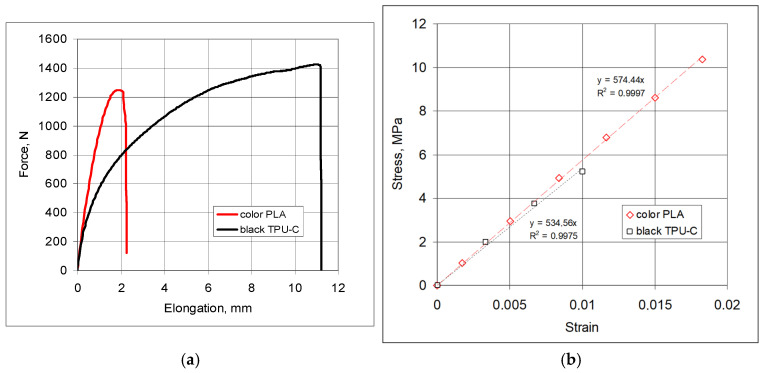
The force-elongation curve for the ligament material *black* and *color* (**a**) and the linear elastic range (**b**).

The core of the present work involves experimental models that have been used to gain a better understanding of the behavior of chiral structures, with particular attention to auxetic behavior. All examined materials have a robust structure achieved through the assembly of printed polymeric unit cells. By assembling separately manufactured cylinders (which constitute the nodes of the structure) and matching ligaments, hexachiral structures were obtained without gluing individual parts of the unit cells: cylinders and ligaments, as shown in Figure 7. The separate manufacturing of ligaments and their subsequent assembly allows for the creation of structures with varying numbers of unit cells. The developed innovative process for assembling chiral structures also allows for adjusting the auxetic properties by altering ligament lengths.

The studied chiral structures were assembled from repeating units with a 6-fold symmetry. The chiral polymer structures were assembled using a press-fit method, ensuring dimensional accuracy for all structural elements. The considered parameters defining the chiral geometry include the radius of the cylinders (nodes) equal to r = 17 mm, the distance between the centers of the cylinders (R = 96 mm), the length of the ligaments (L = 90 mm), the width of the ligaments (b = 25 mm), the wall thickness of the ligaments (t = 1 mm), and the angle between the projected line connecting the cylinders and the ligaments (β = 20.7°)

Flexible ligaments were printed (3D printer, Bambu Lab X1 Carbon, Shenzhen, China) from two types of materials: PLA (PL color, Fiberlex S.A., Brzezie, Poland) and Thermoplastic polyurethane + Carbon fiber (TPU-C black, Fiberlex S.A., Brzezie, Poland). The mechanical properties and the deformation characteristics of the ligament materials obtained from the tensile tests are presented below.

The material of the *black* ligaments exhibited greater elongation under stretching and slightly greater strength; for comparison, the estimated values are 29.1 MPa for the *color* ligaments and 25.1 MPa for the *black* ligaments. In terms of elastic properties, these materials differ in elongation, although the linear range is very limited (Figure 7b). The material of the ligaments marked as *black* exhibits significant *ductility* (Figure 7a).

**Figure 8:**
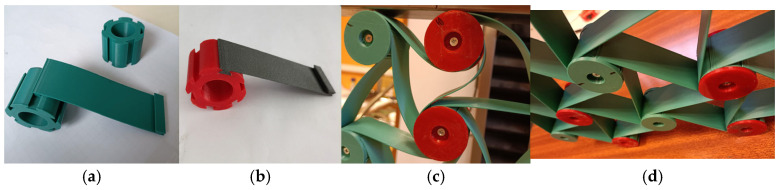
A sample of an assembled hexachiral structure and its individual parts (elements of the structure under construction—(**a**,**b**), view of sections of the structure—(**c**,**d**)).

In the studied structures, each of the six ligaments was tangentially connected to one node (Figure 8c) and secured with a disk. The wall thickness of the cylinders (nodes) and the ligaments was 10 mm and 1 mm, respectively. The angle between adjacent ligaments and the angle between the tangent line and the line connecting the centers of the cylinders (nodes) are θ and β (Figure 3). The studied structures are a combination of rigid (cylindrical nodes) and flexible materials (ligaments).

To characterize the dimensional changes in the structures, measurements were taken of both the outline and the distances L between the centers of the cylinders. Measurements were made directly on the samples compressed in the strength testing machine and with the use of photographic documentation. This allowed for measurement accuracy of 0.1 mm.

### 2.2. Experimental Testing, Uniaxial Compression of 3-4-5-4-3 Structures

To determine the extent to which hexachiral structures are susceptible to instability, a series of compression tests was conducted on various samples, taking into account significant dimensional changes. The scope of these changes corresponded to the loads at which reversible deformations occurred. This ensured that the structures were resistant to cracking and damage.

Carefully assembled structures in four combinations were subjected to compression tests, with an initial analysis focusing on structures possessing a symmetrical arrangement of cylinders (nodes) 3-4-5-4-3. To compare the behavior of these structures, two types of elastic materials were used for the ligaments. In the course of the experiments, the following reactions were measured: stress–strain and the loading and unloading curves. The mechanical properties of the ligaments of the studied structures are presented in Figure 7.

**Figure 9:**
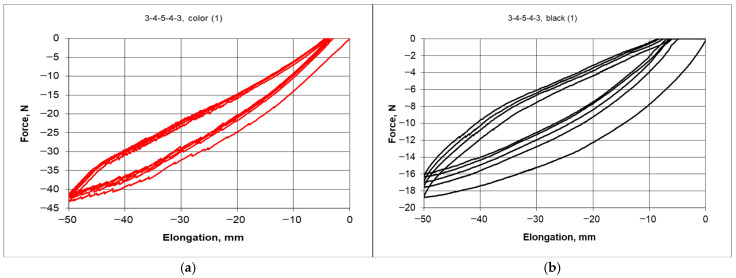
Hysteresis curves for hexachiral structures, with PLA (*color*) ligaments —(**a**,**b**) with *black* Firflex CF ligaments (Fiberlex S.A., Poland).

The stress–strain hystereses (Figure 9) were obtained during cyclic compression of the structures for both types of ligaments differed from each other. The structure with black ligaments was more flexible (lower compressive force) and more ductile (greater strains at zero force)—Figure 9b. The structure built from the color material exhibited a narrower hysteresis, and compressing it by 50 mm required greater force. The occurrence of hysteresis indicates the ability of structures to absorb energy.

The unloading response curve does not follow the same path as the loading response curve, which creates a hysteresis loop. By maintaining a twin-like architecture of the structures, hysteresis was obtained in compression and decompression, differing in load range and width. The differences in the properties of the ligament material lead to varying behavior of structures under compression. Thus, for the black material, compressing to 50 mm required significantly less force. The common feature of these twin structures is that both structures experienced slight plastic deformation due to compression, which increased with each of the next five cycles of compression—Figure 9a,b. The unloading to zero force between each compression indicates a change in dimensions. It follows that after unloading, a small irreversible deformation remained. This deformation for the black material structure was greater than that for the color material structure. This is related to the difference in the properties of the ligament materials, which remained elastic, and no stiffness degradations or other defects and cracks were detected in them.

**Figure 10:**
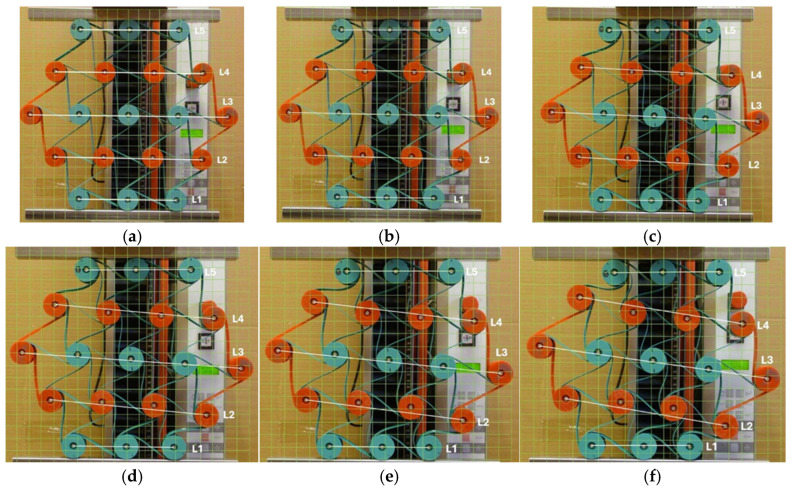
3-4-5-4-3 structure subjected to compression for different compression values (color ligament material, and (**a**) 0 mm, (**b**) 10 mm, (**c**) 20 mm, (**d**) 30 mm, (**e**) 40 mm, (**f**) 50 mm).

The process of cyclic compression of the structure is illustrated in the photographs in Figure 10.

In the hexachiral structure, each of the six ligaments was tangentially connected to one node. In the investigated 3-4-5-4-3 structure, there are 19 cylindrical nodes, 7 of which possess a complete set of 6 ligaments. The 7 nodes are located within the structure and form complete hexachiral unit cells. There are 12 remaining cylindrical nodes with 3 ligaments each. The incomplete unit cells create a surrounding zone for the complete unit cells.

During uniaxial compression, the outer unit cells are subjected to different loading conditions, resulting in irregular deformation of the structure. Among the 12 cylindrical nodes interconnected by three ligaments each, several types of deformation can be distinguished. The cylindrical nodes in layers L1 and L5 (Figure 10) undergo rotation and wrapping of the ligaments, while the outer nodes in layers L2 and L4, as well as in layer L3, keep roughly the same distance from each other, and the ligaments tangent to them become stretched. Although the structure has undergone compression both vertically and horizontally, the resulting dimensional changes in the individual layers vary. The smallest horizontal contraction for the L3 layer yields the lowest negative values of the Poisson’s ratio.

Initially angle between the adjacent ligaments was θ = 60°. Cylindrical nodes separated by a distance R initially arranged themselves in parallel layers L. The initial distance between the layers was Rcos θ. As a result of compression, the structure underwent contraction, and the corresponding Poisson’s ratio was calculated as the ratio of the relative changes in the lengths of segments L to the relative change in the height of the structure (Figure 10a–d).

When the structure was compressed by 10 mm (approximately 3%), the dimensional changes in the individual layers remained visually uniform. The bending of the ligaments and the rotation of the nodes were relatively similar—Figure 10a. As the degree of compression increased, a stronger deformation occurred, characterized by changes in the ligament shape and the bending of individual layers. When compressed to 50 mm, not only were there significant differences in the contraction of section L, but, more importantly, varying degrees of bending and wrapping of the ligaments around the nodes. Figure 10b–e.

The photographic documentation illustrates the deformation of structures with gradual strains, precisely depicting the response and deformation of unit cells during the compression of structures at successive stages. The compressed structure undergoes a twist to the right, while the gaps between the cylindrical central layers remain nearly parallel. The outer cylindrical nodes are wrapped by the ligaments in the upper left side and lower right layer, drawing closer towards each other. As a result, there is a significant dimensional change in the ligaments in the upper and lower layers of the structure (L1 and L5), as well as distinct coiling around the cylindrical nodes.

The presented results for the 3-4-5-4-3 color structure particularly highlight the non-uniform shifting of the nodes and the lines L during the compression of the structure (Figure 10). More favorable behavior of structures under compression was achieved after introducing minor modifications. By shifting the anchor points of the external ligaments, a new type of structure is obtained that exhibits different behavior under compression. First and foremost, this structure acquires an internal stress state. This state arises due to the adjustment of the flexible ligaments, which change from straight to an arch-like shape.

**Figure 11:**
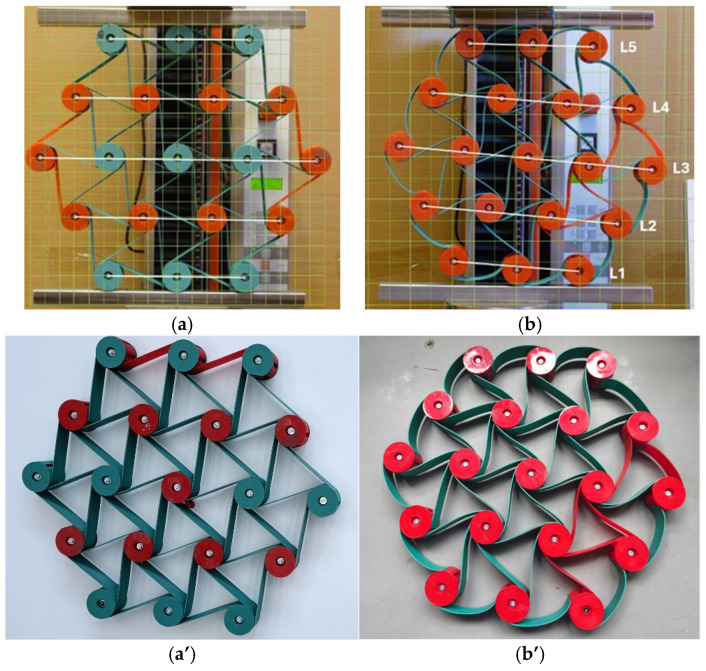
Comparison of the 3-4-5-4-3 structure in its original version (**a**,**a’**) and after modification (**b**,**b’**) (color ligament material).

**Figure 12:**
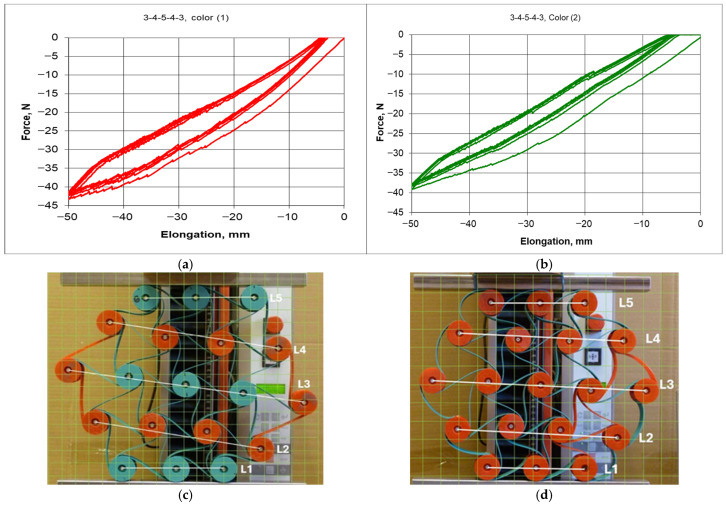
Comparison of the hysteresis of the 3-4-5-4-3 color structure in its original version (**a**) and after modification (**b**), as well as photographs of structures (**c**) compressed by 50 mm (**d**) (color ligament material).

In such a modified structure, the internal forces of one part of the structure act on another part of that structure. The new modified “chiral flower” structure—Figure 11b, exhibited a different response to axial compression. This is particularly evident considering not only the narrower hysteresis loop (Figure 12a,b), but also the image of the compressed structures of 50 mm—Figure 12c,d.

Not only did the hysteresis in the force-deformation system become narrower, but also the auxiliary lines L were almost in parallel during compression. A smaller hysteresis indicates a greater stiffness of the structure, which can be correlated to its internal stress state. The parallel behavior of the lines L during compression indicates a more uniform displacement of the layers in the structure.

**Figure 13:**
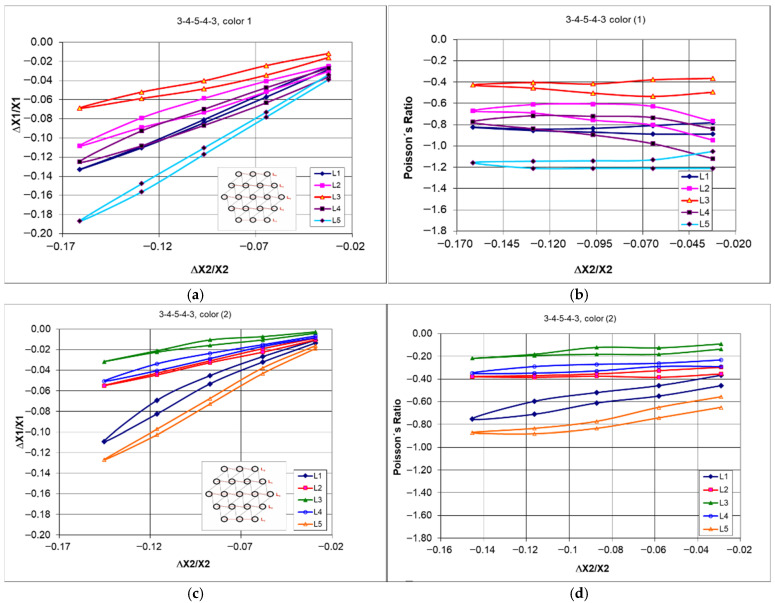
Comparison of the behavior of the 3-4-5-4-3 color structure under cyclic compression in the original version (**a**,**b**) and after modification (**c**,**d**).

Thus, the results of dimensional changes and the reduced spread of the Poisson’s ratio indicate the benefits yielded by introducing this modification—Figure 13.

However, considering the values of the Poisson’s ratio, the varying behavior of the individual layers of the structure has been confirmed Figure 13. With uniform changes caused by compression, the obtained values of the Poisson’s ratio for individual layers should be closely matched.

Differences in the behavior of compressed structures shown in Figure 12c,d lead to significant differences in dimensional changes and in the Poisson’s ratio values. The latter go below minus one (−1)—Figure 13d.

Nevertheless, one more characteristic remains relevant in both unmodified and modified types of structures. It involves the large deformations of the bottom layer of the structure (L1) and small deformations of the middle layer under axial compression of the 3-4-5-4-3 structure.

**Figure 14:**
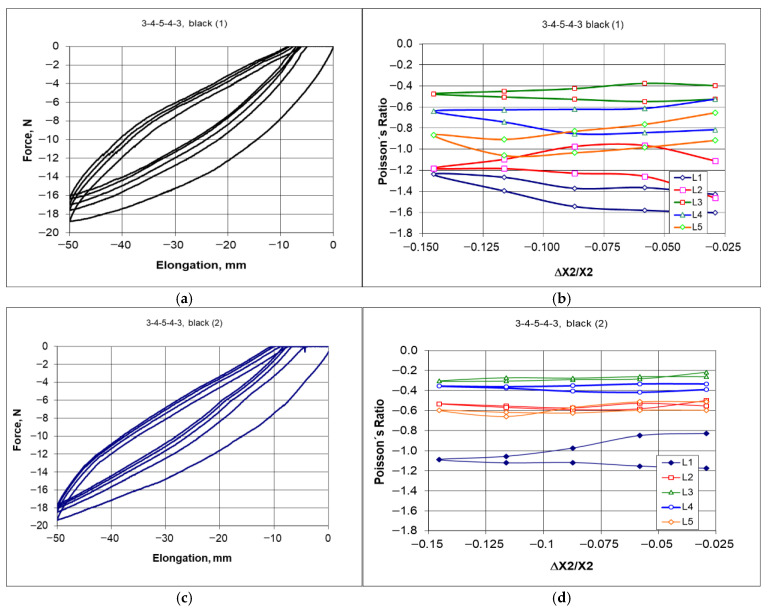
Comparison of the behavior of the 3-4-5-4-3 black structure under cyclic compression in the original version (**a**,**b**) and after modification (**c**,**d**).

Similar results were obtained for the twin structure, with ligaments made from the black material. The behavior as described above also applies to the 3-4-5-4-3 black structure. The confirmation of this can be seen in Figure 14.

The modified 3-4-5-4-3 black structure, in its original and modified form, subjected to compression by 50 mm and the dependency of Poisson’s ratio on the amount of compression correspond to dimensional changes in the individual layers (Figure 14). In this case, likewise, the modified structure resulted in a narrower hysteresis shape (Figure 14a,c) and a smaller spread of the Poisson’s ratio values (Figure 14b,d).

Although the ligament material is more ductile in this case, the results obtained from the compression tests of the structure are similar to the previous ones. However, the calculated values of the Poisson’s ratio exhibit a greater variance. In this case, as well, the selected degree of compression of the structure generates a characteristic distribution of deformations throughout the entire structural network.

In summary, it should be emphasized that only this type of measurements of dimensional changes at specified intervals (10 mm during compression up to 50 mm) for individual unit cell layers (denoted as L1, L2, L3, L4, and L5—Figure 10) provides the data that allow for a closer understanding of the auxetic behavior of hexachiral structures. The presented results indicate significant differences in the values of the Poisson’s ratio obtained for the individual layers, with more varied results for structures with ligaments from the black material. Additionally, it is vital to note that the Poisson’s ratio values show little dependence on the level of deformation (ΔX2/X2).

The variability range of this parameter for the studied structures is relatively large, spanning from −0.4 to −1.2 for the color material structures and from −0.4 to −1.6 for the black material structures. The estimated standard deviation of these quantities is approximately 0.3. However, the value of the Poisson’s ratio calculated for the structure outlines under 50 mm compression was −0.47 (with a shrinkage of 14.5%). This value is an important design parameter; however, it does not reflect the process of compression.

The presented relationships indicate that with a significant variation in the Poisson’s ratio, there is no influence of the degree of deformation, and that the calculated values based on the changes in the dimensions of the outline fall within the given range. Thus, the obtained negative values of the Poisson’s ratio are close to −1.

As a result of the stresses acting within the material of the elastic ligaments, the modified structure becomes stiffer (narrower hysteresis—Figure 14c). The internally acting forces combined with external loads provide a more uniform picture of dimensional changes during compression. The accumulation of residual stresses and working compressive loads is also evident in this case.

After modification, the hitherto static ligaments of the structure are subjected to bending stresses (tension and compression) while the nodes, influenced by the ligaments, undergo rotation. The dimensional changes in structures resulting from elastic deformations become more uniform in this case.

### 2.3. Experimental Testing, Uniaxial Compression of 3-4-3, 3-4-3-4-3, and 3-4-3-4-3-4-3 Structures

The influence of the number of unit cells on the auxetic properties was examined for structures with the following configurations: 3-4-3, 3-4-3-4-3, 3-4-3-4-3-4-3. The proposed modular method for assembling the structure enables a mass construction of elaborate systems. The designed geometry of the structures, achieved through a gradual increase in their size, facilitated the observation of dimensional changes, particularly the occurrence of specific deformations. The assembled structures were subjected to compression using a testing machine. The results of the tests are presented both in the form of photographic documentation and force-deformation curves.

The dimensional changes were monitored through both direct measurement and photographs.

**Figure 15:**
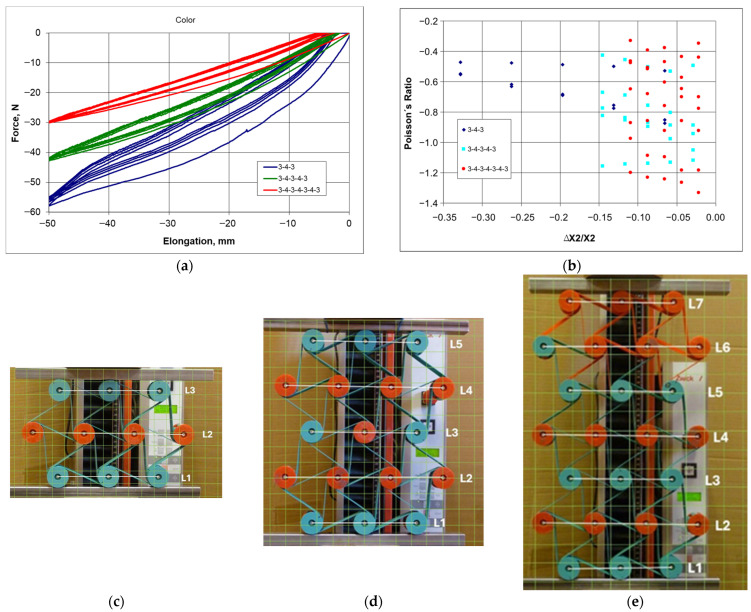
Deformation as a function of compressive force for 3-4-3, 3-4-3-4-3, and 3-4-3-4-3-4-3 structures (**a**) and the values of Poisson’s ratio—(**b**), along with photographs of the structures with highlighted dimensions L—(**c**–**e**) (color ligament material).

Based on this, the values of the Poisson’s ratio were calculated. The relationship presented in Figure 15b indicates that for the studied structures, its values vary widely, i.e., from about −0.2 to about −1.4.

The presented hysteresis (Figure 15a) was created as a result of 5 cycles of compression and decompression at a speed of 10 mm/min, with an amplitude of 50 mm, corresponding to the elastic properties of the ligament material. Although as a result of compression, the structures underwent a slight plastic deformation, which amounted to 3.2 mm (H = 186 mm), 2.4 mm (H = 344 mm), and 2.2 mm (H = 491 mm), respectively, for structures 3-4-3, 3-4-3-4-3, and 3-4-3-4-3-4-3.

The size of the structure clearly influences the shape and width of hysteresis in the contraction-force curve, with a compression of 50 mm requiring a greater force for a taller structure than for a shorter one (Figure 15a). Thus, the width of the obtained hysteresis decreases as the structures become more complex. This observation seems obvious, as with the extension of the structure, the specified change in vertical dimensions is easier to obtain.

The resting state (initial) configurations shown in Figure 15c–e have the distances between the centers of the cylindrical nodes marked with white lines. During compression, these distances decrease, which happens in various ways. The smallest dimensional changes occur in the central unit cells, which leads to the least negative values of the Poisson’s ratio.

It should be noted that the dimensional changes in the studied structures resulting in the relationship ΔX1/X1 vs. ΔX2/X2 exhibit an almost linear behavior, with its slope corresponding to Poisson’s ratio. With vertical compressive strain, there is a linear increase in the angle of deformation in the horizontal direction. Upon a detailed analysis of this tendency observed in the studied structures, one can find peculiar behavior of individual layers and discrepancies in the compression levels. The particularly characteristic behavior of some structures arises from the interaction mechanism between individual unit cells. For the 3-4-3 structure (Figure 15c), the unit cells in layers L1 and L3 were the most compressed. The middle layer (L2) was the least deformed. In more intricate structures, various unusual deformations were observed. In the structure 3-4-3-4-3 (Figure 15d), the central layer (L3) was the most compressed, while layers L4 and L2 were significantly less affected. The placement of layer L3 between the longer layers L4 and L2 was crucial in this case. Similarly, in the 3-4-3-4-3-4-3 structure (Figure 15e), the shorter L3 and L5 layers undergo the greatest compression, while L4 and L6 layers undergo the smallest compression. The greatest compression corresponds to the smallest values of the Poisson’s ratio. During compression in these complex structures, the greatest contraction occurs in the middle layers, whereas the smallest contraction occurs in the outer ones.

From this comparison, it follows that the ligaments in the first lower layer and the third middle layer undergo the most irregular deformation (Figure 15b,c).

The mechanical response of hexachiral structures to an applied external force, at a given amplitude of cyclic compression, shows that the internal unit cells are compressed more strongly than the external ones. This indicates that the structures exhibit graded deformation dependent on their position.

**Figure 16:**
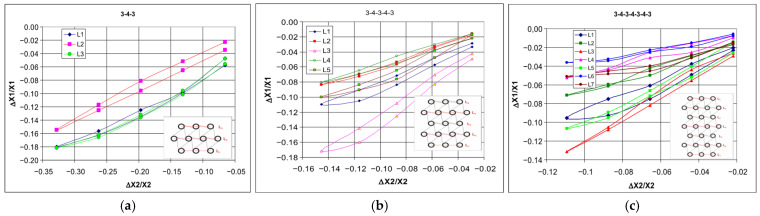
Change in dimensions of 3-4-3 (**a**), 3-4-3-4-3 (**b**), and 3-4-3-4-3-4-3 (**c**) structures during compression based on measurements of L segments.

An experimental comparative study of three selected types of hexachiral structures allows for obtaining a realistic picture of compression. Although the studied chiral structures exhibit similar yet distinctive types of hysteresis in the strain-force relationship, their gradual deformations under uniaxial compression vary (Figure 16).

Nevertheless, they display lower values of the Poisson’s ratio than the theoretical value of minus one (−1).

### 2.4. Experimental Testing, Multi-Directional Compression of 3-4-5-4-3 Structures

A compressed hexachiral structure may serve as a component for storing elastic energy. The energy storage mechanism involves the flexible bending of ligaments and their wrapping around the cylindrical nodes (cores). Such multi-directional compression, analogous to pseudo-isostatic compression, can be a prerequisite for homogeneous deformation of the structure, which can achieve the theoretical value of the Poisson’s ratio.

**Figure 17:**
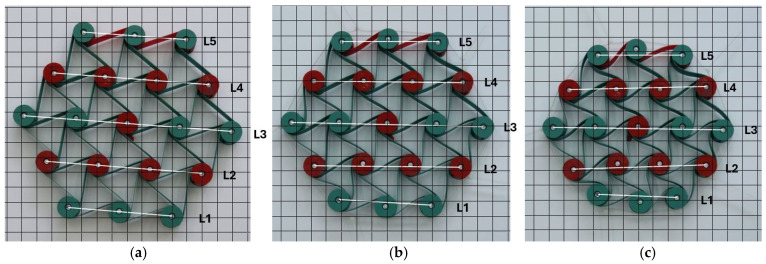

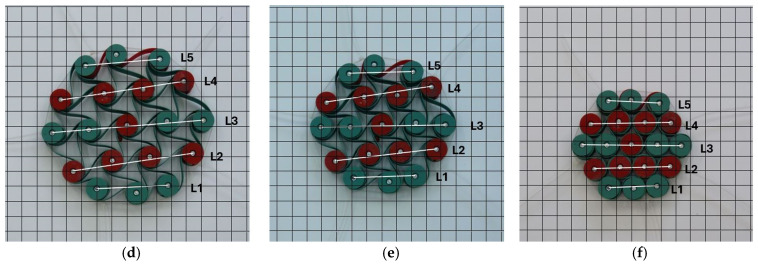
Stages of multi-directional compression of 3-4-5-4-3 structures (**a**–**f**) (color ligament material). The notations L1–L5 refer to the lines connecting the centers of the nodes in each row.

**Figure 18:**
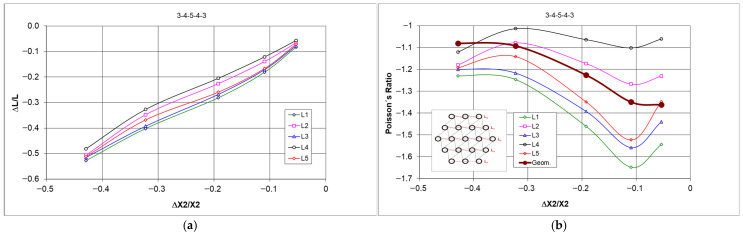
Change in dimensions of the structure (**a**) and the value of the Poisson’s ratio (**b**) as a function of relative contraction.

The subsequent stages of multi-directional compression of the structure (Figure 17) can be treated in approximation as uniform compression, which, as shown in Figure 18, does not produce the theoretical value of the Poisson’s ratio (−1). Considering the dimensional changes in its individual parts, smaller values of this parameter are obtained.

The obtained relationships between ΔL/L vs. ΔX2/X2 follow a curve, indicating an unusual behavior in the compression process—Figure 18a. The presented relative changes in the dimensions of the L segments are parallel for small contractions, which is no longer the case for larger changes. The complete contraction yields a parallel arrangement of the auxiliary lines (Figure 17f). The behavior of this structure arises from its specific malleability achieved under the influence of external forces.

Theoretically, for hexachiral structures with m = *n* = 5, the value of the Poisson’s ratio is ν = −1.02 (r/R = 0.28), which in this case corresponds to the maximum compression of the structure. What is especially interesting is the experimentally established dependence of Poisson’s ratio on the level of contraction. For uniaxial compression, such a relationship did not occur.

For comparison, multi-directional compression tests were conducted on auxetic structures with *black* material ligaments.

Thus, in this case, with increasing deformation, the ligaments underwent smooth bending and wrapping without breaking or buckling.

**Figure 19:**
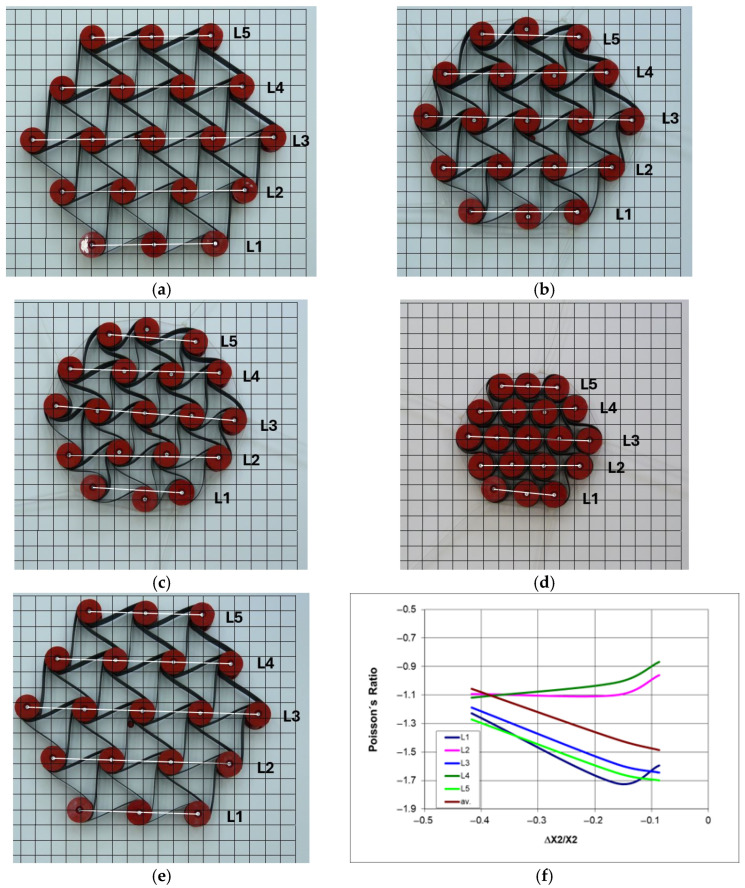
The subsequent stages of multi-directional compression of the 3-4-5-4-3 structure with black material ligaments (**a**–**e**), and the relationship of the Poisson’s ratio with the change in dimensions—(**f**) (black ligament material).

While the photographs b, c, and d in Figure 19 pertain to the subsequent compression stages, photograph a illustrates the structure in its original dimensions, whereas image e shows the dimensions of the structure directly after unloading. The difference in linear dimensions compared to the initial dimensions is approximately 4.5%. Reduced stiffness of the ligaments resulted in greater deformations of the structure after compression. It can be observed (Figure 19) that some internal ligaments of the structure bend into the shape of the letter S (serpentine shape), while the external ligaments curve, resembling the shape of the letter C (catenary shape).

The behavior of the Poisson’s ratio during compression, shown in Figure 19f, is particularly noteworthy. The outer layers (L1 and L5) are compressed the most, as they exhibit the lowest values of the Poisson’s ratio. In contrast to this, for layers L2 and L4, the Poisson’s ratio takes on the least negative values. The longest middle layer (L3) behaves similarly to the outer layers L1 and L5, as it reaches similar values of the Poisson’s ratio.

Figure 19d illustrates the maximum compression of the structure, corresponding to a value of >45%. Cylindrical nodes come into contact, resulting in a state of compaction. In the case of multi-directional compression, when the compressive force acts directly and (theoretically) uniformly on the cylindrical nodes, the structure undergoes contraction, and the corresponding Poisson’s ratio approaches minus one. In the actual case of multi-directional compression, there is a lack of uniform deformation, and the middle layer L3 of the structure experiences the least contraction. The difference in behavior of individual layers during multi-directional compression is reflected in the local values of the Poisson’s ratio; approximately −1 for L3 and about −1.5 for the outer layer L1. This may indicate varying stresses in different parts of the structure.

The above relationships indicate that the applied method of multi-directional compression does not ensure uniform contraction, and the determined value of the Poisson’s ratio differs from the theoretical one. One must add that this type of stress on chiral structures can be considered an example of a unique energy storage mechanism. While the utilized clamping mechanism offered a limited degree of stopping cylinder rotation, it yielded consistent characteristic outcomes in the shifts in the Poisson’s ratio during compression.

This mechanical manipulation, involving the compression of chiral structures from all sides, can be treated as a potential proposal for application in technology.

## 3. Discussion

The auxetic properties of a chiral structure were first determined analytically. The obtained general relationship indicates that the value of the Poisson’s ratio depends both on the geometric parameters: the diameter of the cylindrical nodes r and the distance between the cylinders R, as well as on the size of the structure, i.e., the number of unit cells horizontally and vertically.

It is well established that increasing the r/R value affects the reduction in the Poisson’s ratio [3], which has been confirmed here.

In research, the thickness of ligaments typically does not change, but their length or type of material often varies. The analysis did not take into account the ligament thickness and assumed this parameter to be constant. The theoretical values of the Poisson’s ratio for hexachiral structures are slightly more negative than −1. In comparison, the theoretical dependence presented in another study [46] and capturing the thickness of the ligaments is also close to −1, but slightly below it (less negative) (Figure 4).

A key theoretical insight from the geometry-properties relationship is that under uniaxial compression, the value of Poisson’s ratio remains independent of the degree of deformation. That is especially since it has been confirmed by the experiments using the testing machine.

The value of this parameter can be altered only by changing the geometry. The problem of maintaining a stable Poisson’s ratio is an important issue raised in the literature [51,53,54,55,56]. The presented analyses and experimental studies of uniaxial compression show that this can be achieved for hexachiral structures.

The unusual behavior of hexachiral structures is confirmed in direct observation. Through experimental studies of hexachiral structures, the present work has demonstrated the ability of such mechanical metamaterials to undergo directional internal deformation under the influence of external forces. Due to their unique structural asymmetry, structures exhibit complex deformation behavior, including a coupling of bending and twisting that is not easily predictable.

Observations indicate that significant deformations occur in the lower outer layer of the structure (L1), while unit cells in the middle layers undergo minor deformations, as seen in the observations of axial compression of a 3-4-5-4-3 structure. The stronger compression of the central unit cells results from the cumulative interaction of neighboring cells. This type of deformation can be compared to the standard deformation patterns of the behavior of structures made from cylindrical unit cells subjected to compression [57]. According to these studies, the onset of sequential collapse of unit cells during the compression of cellular structures relates to the deformation of the central internal unit cell layer.

Thanks to experimental observations and the accumulation of a sufficiently large body of results, it is possible to draw some general conclusions. Observations indicate that the mechanism of compressing the hexachiral structure involves bending the ligaments, which forces the rotation of nodes and consequently leads to the ligaments wrapping around them. Such behavior of the structure is referred to as the chiral effect.

By employing both direct measurement and photographic documentation, it was possible to accurately track the dimensional changes occurring in individual unit cells during the compression of structures. Based on this, one can distinguish the displacement patterns between parts of unit cells, as well as the mutual spacing of cylindrical nodes and changes in ligament curvatures. By precisely defining the dimensional changes in both horizontal and vertical directions, as well as the changes in the outline dimensions of the structure, a complete and consistent set of Poisson’s ratio results was obtained.

First and foremost, it was determined that the hexachiral chains (layers) contract unevenly, and the cylindrical nodes rotate to varying degrees. As the internal structure of the ligaments bends into a wave-like shape, the external ligaments form arch-like segments.

The results of the observations presented in the photographs—Figure 10, Figure 11 and Figure 16—show that the ligament structures undergo various deformations when deformed, with defects and damage to the structures occurring only at much higher compression amplitudes. Limiting the amplitude to 50 mm (for 340 mm structures), no cracks were found, although compression tests on the same ligaments were repeated many times.

Due to local deformations, the properties of auxetic structures change locally, which has already been noted in previous studies [58,59].

In the results of cyclic compression and decompression, relationships of contraction and force were obtained, which take the form of stable hysteresis. It is difficult to justify the occurrence of a hysteresis for a purely elastic range. However, the presence of the plastic component, especially for ductile materials, allows for compliance with the principles of mechanics. In each of the examined cases, it was found that after unloading, the dimensions of the samples slightly decreased. The structures, when unloaded, exhibited minimal plastic deformation. It was noted, in connection with the structures with black ligaments (ductile material), that they lost the residual deformation over time.

The occurrence of a complete loading-unloading loop has already been observed in many metamaterial structures subjected to cyclic loading [24,60,61].

The recoverability slightly decreased after unloading when the sample was subjected to a larger, centrally applied, concentrated load, likely due to plastic deformation. Thus, the hysteresis interpreted with respect to the viscoelasticity of the material contributes to energy dissipation. It can be stated that the samples exhibited nonlinear stiffness. The repetitive change in the behavior of hysteresis in load–displacement curves was also observed mainly at low displacement values, with the presence of static friction blocking deformation [60], and through the emergence of micro-cracks [16,24,61,62]. It is indicated that, due to deformation, structures made from polymer materials acquire residual displacements, which can be attributed to plastic deformation.

The effects of multi-directional compression described herein represent a novel example of auxetic behavior, with potential application significance.

One can compare this process to the clockwise twist mentioned in one review work [44]. Mechanical metamaterials under compression using a clamp serve as a reservoir with a recoverable elastic energy of a high density—proposed as a unique energy storage mechanism [3].

**Figure 20:**
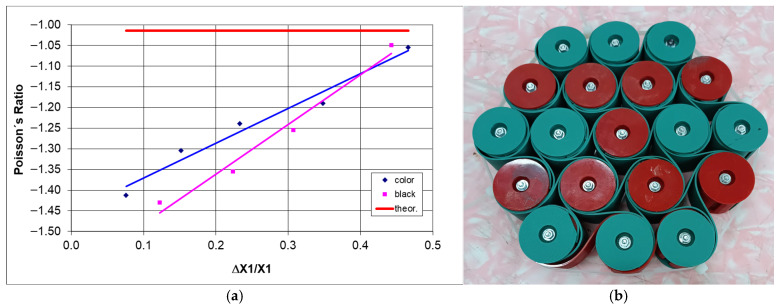
Poisson’s ratio as a function of strain degree for hexachiral structures 3-4-5-4-3 subjected to multi-directional compression in the plane—(**a**), dense structure—(**b**).

While uniaxial compression of hexachiral structures, both theoretically and experimentally, does not yield the dependence of Poisson’s ratio on the degree of deformation, multi-directional (pseudoisostatic) compression in the plane does. Figure 20a presents the changes in the Poisson’s ratio for the 3-4-5-4-3 structures under multi-directional compression, obtained from the measurements of the outline of the structures.

The presented dependence shows the trends of the Poisson’s ratio of multi-directionally compressed structures in the plane, which was previously illustrated in Figure 19b,f.

Due to the forced contraction of structures in all directions within the plane, the ratio of relative dimensional changes, known as the Poisson’s ratio, varies monotonically with contraction. The structure image obtained after maximum densification (Figure 20b) corresponds to the theoretical projections. It can be added that the described density cannot be realistically achieved under uniaxial compression.

It is important to emphasize that the literature describes numerous successful simulations of chiral structure behavior, e.g., [19,29,45,48,63]. However, these simulations still require further experimental validation to create real structures for functional application. The approach presented in this work, involving the assembly of structures and the analysis of their actual behavior, is aimed at this very goal.

## 4. Conclusions

The article has presented a systematic study of two-dimensional hexachiral structures composed of rigid, cylindrical nodes, connected by tangent elastic ligaments. The proposed process for manufacturing structures in the form of composites represents a feasible and straightforward research task concerning the properties of such structures with unconventional geometric features. The practical implementation of chiral metamaterials at any scale can be achieved according to the demonstrated process.

Artificial materials of this kind exhibiting auxetic behavior can be used to create parts of engineering structures with variable dimensions and a potential for storing elastic energy or, for example, in metamaterial machines [64].

It has been observed that the modes of deformation observed in these structures are bending of the ligaments and forced rotation of the nodes. The results of the experiments confirm the highly nonlinear response of structures to cyclic compression, stemming from material and geometric effects at large displacements. Both the elastic PLA material and the ductile TPU-C material (with carbon fibers) exhibited hysteresis in their mechanical properties within the elastic range, consistently showing slight residual plastic deformation. Both the type of ligament material and the number of unit cells influenced the auxetic behavior of the structures and the values of Poisson’s ratio.

The developed approach to studying metamaterial structures, combining geometric analysis, fabrication of real structures, and experimental measurements, may be appealing for design solutions in various fields of engineering. The challenge formulated in this way may serve as an encouragement for further exploration and work on artificial materials (metamaterials) that can meet the demands in various areas of technology.

## Data Availability

The original contributions presented in this study are included in the article. Further inquiries can be directed to the corresponding author.
